# Exosomes for Wound Healing: Purification Optimization and Identification of Bioactive Components

**DOI:** 10.1002/advs.202002596

**Published:** 2020-10-27

**Authors:** Britta F. Hettich, Maya Ben‐Yehuda Greenwald, Sabine Werner, Jean‐Christophe Leroux

**Affiliations:** ^1^ Institute of Pharmaceutical Sciences Department of Chemistry and Applied Biosciences ETH Zurich Zurich 8093 Switzerland; ^2^ Institute of Molecular Health Sciences Department of Biology ETH Zurich Zurich 8093 Switzerland

**Keywords:** CD73, exosomes, human bone marrow stromal cell line, lipids, wound healing

## Abstract

Human mesenchymal stem cell exosomes have been shown to promote cutaneous wound healing. Their bioactivity is most often attributed to their protein and nucleic acid components, while the function of exosomal lipids remains comparatively unexplored. This work specifically assesses the involvement of lipids and the transmembrane enzyme CD73 in the exosomes’ biological activity in stimulating the cutaneous wound healing process. Since exosome preparation processes are not harmonized yet, certain production and purification parameters are first systematically investigated, enabling the optimization of a standardized protocol delivering high exosome integrity, yield, and purity. An in situ enzymatic assay is introduced to specifically assess the vesicle functionality, and quantitative proteomics is employed to establish the cell culture conditions yielding a stable exosome protein profile. Using a combination of in vitro approaches, CD73 and constitutional lipids of HPV‐16 E6/E7 transformed human bone marrow stromal cell‐derived exosomes are identified as key bioactive components promoting the exosome‐driven acceleration of processes required for wound repair. A pilot wound healing study in mice indeed suggests a role of lipids in the exosomes’ biological activity. Strikingly, the extent of the bioactivity of these exosomal components is found to be dependent on the target cell type.

## Graphical abstract

Stem cell exosomes harbor an intrinsic therapeutic potential that may be beneficial in promoting the skin wound healing process. This study specifies the optimal production and purification conditions to obtain high recoveries of bioactive exosomes from transformed human bone marrow stromal cells and analyzes the functional role of exosomal lipids and the transmembrane enzyme CD73 in their wound healing‐promoting activity.

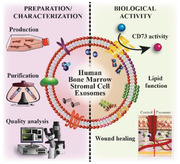

## 1 Introduction

Cell‐secreted extracellular vesicles (EV) have been identified as key players in intercellular communication processes.^[^ ^1^, ^2^ ^]^ Exosomes are a therapeutically relevant EV subtype given their activity in a broad range of disease categories, including immune‐related disorders and tissue regeneration.^[^ ^3^, ^4^ ^]^ They correspond to a heterogeneous population of naturally occurring, endosome‐derived nanovesicles with a maximal diameter of 200 nm, and are constituted by a protein‐containing lipid bilayer, which encapsulates nucleic acids, proteins, and other bioactive components.^[^ ^1^, ^2^, ^5^, ^6^ ^]^ Exosomes can be viewed as cellular fingerprints, as their composition is highly dependent on the producing parent cell, and dictates the biological processes these natural vesicles can trigger at target sites.^[^ ^1^, ^2^, ^7^ ^]^ In contrast, certain proteins’ absence or presence can be used as markers of exosome identity and purity, independent of the cell source.^[^ ^1^, ^2^ ^]^

Exosomes derived from human mesenchymal stem cells (hMSC) have been sporadically reported to improve cutaneous wound healing through the modulation of inflammation, proliferation and/or matrix remodeling.^[^ ^3^, ^4^, ^8^ ^]^ Certain proteins and microRNAs carried by the exosomes have so far been proposed as key mediators of these effects.^[^ ^9^, ^10^, ^11^ ^]^ The transmembrane enzyme ecto‐5′‐nucleotidase (CD73) expressed by hMSC exosomes has been associated to their beneficial immune‐modulatory function in graft‐versus‐host‐disease^[^ ^12^ ^]^ and capability to promote cartilage repair,^[^ ^13^ ^]^ also suggesting a potential role for CD73 in the hMSC exosome‐driven acceleration of skin wound healing. Furthermore, a previous study identified exosomal lipids as key components of the exosome effects in anticancer therapy.^[^ ^14^ ^]^ Indeed, certain lipid classes (e.g., sphingolipids, glycerophospholipids) can exert immune‐modulatory, mitogenic, migratory, or angiogenic effects, which have been proven beneficial in impaired cutaneous wound healing.^[^ ^15^, ^16^, ^17^ ^]^ The function of specific lipids in mediating the exosomal therapeutic efficacy was, however, hardly investigated in past studies.^[^ ^18^ ^]^

Currently, progress in exosome research is in part hampered by the heterogeneity in the applied preparation procedure.^[^ ^19^, ^20^ ^]^ The significant variations in the purification efficiencies of the different isolation methods (i.e., the enrichment of exosomes compared to other EV and impurities) prevents, in most cases, the generalization of findings across different studies.^[^ ^20^, ^21^, ^22^ ^]^ To date, an array of different state‐of‐the‐art exosome preparation strategies is available and a growing number of publications has investigated their assets and drawbacks.^[^ ^6^, ^23^, ^24^ ^]^ Ultracentrifugation (UC) was the first exosome isolation method to be established and although it remains the gold standard,^[^ ^25^ ^]^ drawbacks such as copurification of protein aggregates, morphological changes (e.g., agglomeration, membrane rupture), and tedious processing times have motivated the implementation of faster alternatives.^[^ ^6^, ^24^ ^]^ Ultrafiltration (UF) is a more rapid purification method with a proposedly higher purification efficiency,^[^ ^23^, ^26^ ^]^ which when in combination with gravitational size exclusion chromatography (SEC) can produce high yields of pure and intact exosomes.^[^ ^27^, ^28^, ^29^ ^]^

In this work, the biological activity of HPV‐16 E6/E7 transformed human bone marrow mesenchymal stromal cell (HS‐5)‐derived exosomes on different aspects of cutaneous wound healing was investigated, focusing on the role of previously largely unexplored exosomal components, inter alia CD73 and exosomal lipids, in mediating the exosomes’ regenerative capability. To this end, a standardized purification protocol was first optimized to reproducibly obtain intact and bioactive HS‐5 exosomes in high purity and yield.

## 2 Results

### 2.1 Exosome Purification by UC Outperforms UF‐SEC

The purity and functionality of exosomal formulations is highly dependent on the exosome isolation method.^[^ ^20^, ^21^, ^22^ ^]^ In order to obtain a high yield of enzymatically active exosomes, which can be reproducibly used for in vitro and in vivo wound healing studies, the HPV‐16 E6/E7 transformed human bone marrow mesenchymal stromal cell line HS‐5 was selected for a detailed investigation of two common purification strategies. Exosomes were produced by HS‐5 cells for 24 h under serum‐free conditions in order to obviate a possible contamination with serum‐derived components such as EV, lipids, and RNA.^[^ ^1^, ^23^ ^]^ Vesicles were subsequently isolated by either UC at 150 000 × *g* or UF‐SEC and the isolated fractions were characterized according to the recommendations of the International Society for Extracellular Vesicles.^[^ ^30^, ^31^ ^]^

**Figure 1:**
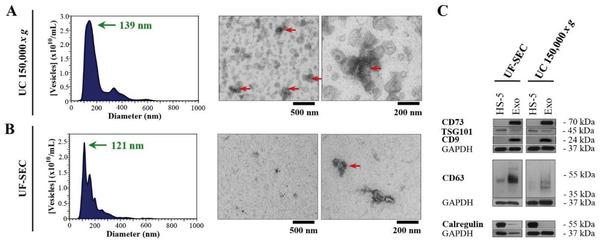
UC purification yields a higher exosome recovery and purity than UF‐SEC. A–C) HS‐5 exosomes isolated by either A) UC at 150 000 × *g* or B) by UF‐SEC after a 24‐h production period. A,B) NTA size profiles (left panels) and TEM images (right panels) of exosomes. Green arrows indicate the main vesicle population with their modal diameter and red arrows indicate vesicle aggregates (panels (A) and (B)). C) Western blot analysis of the exosome marker proteins TSG101, CD9, CD63, and CD73, as well as the contamination marker calregulin, with GAPDH as loading control. Experiments (left panels in (A) and (B); panel (C)) were performed in triplicates and a representative plot/image is shown.

With 5.4 ± 1.8 × 10^10^ vesicles mL^−1^, UC resulted in an approximately twofold higher vesicle recovery than UF‐SEC, which yielded 2.9 ± 1.5 × 10^10^ vesicles mL^−1^. As measured by nanoparticle tracking analysis (NTA), the UC‐isolated vesicles had a modal diameter of 139 ± 6 nm, while those obtained by UF‐SEC were smaller, measuring 121 ± 6 nm (**Figure** **1**A,B). Further vesicle/aggregate populations ranging up to 700 nm were detected with both purification approaches, albeit in low proportions. The size distributions of UC‐ and UF‐SEC‐purified exosomes obtained by NTA were confirmed by transmission electron microscopy (TEM), which also revealed the presence of aggregates in both cases (Figure 1A,B; Figure S1A,B, Supporting Information). Moreover, the vesicles harvested by either isolation method exhibited a distinct cup‐shaped morphology, which has been previously reported as an artefact of the preparatory fixation process required for TEM.^[^ ^23^ ^]^

Immunoblotting revealed that both isolates were enriched in the exosome marker proteins TSG101, CD9, CD63, and CD73 (Figure 1C). The UC preparation was devoid of the endoplasmic reticulum marker protein calregulin, a representative contaminant, whereas the UF‐SEC sample was not. Higher impurity levels following UF‐SEC were also reflected in a higher protein content (161 ± 97 µg protein mL^−1^ for UF‐SEC vs 95 ± 20 µg protein mL^−1^ for UC) and a reduced vesicle‐to‐protein ratio (1.9 ± 0.3 × 10^8^ vesicles µg^−1^ for UF‐SEC vs 5.6 ± 1.2 × 10^8^ vesicles µg^−1^ for UC). The latter has been previously proposed as a measure of the vesicle purity.^[^ ^26^, ^32^ ^]^

Moreover, the CD73 enzymatic activity following UC isolation was tenfold higher than that after UF‐SEC (4.4 ± 0.7 U mg^−1^ for UC vs 0.4 ± 0.1 U mg^−1^ for UF‐SEC, whereby 1 unit (U) is defined as 1 µmol min^−1^). This could indicate a partial activity loss upon the UF‐SEC procedure or, in line with Western blot analysis, a lower purity of the UF‐SEC sample (i.e., less CD73 per isolated µg of protein).

Taken together, the vesicle characteristics (i.e., size, morphology and presence of exosome protein markers) were well inside specifications for exosomes,^[^ ^30^, ^31^ ^]^ demonstrating the enrichment of this EV subtype by both isolation approaches. In contrast to UF‐SEC, UC was able to purify enzymatically active HS‐5 exosomes in a more reproducible fashion, and was hence retained as purification strategy in subsequent experiments.

### 2.2 Preparation Conditions Affect the Exosome Characteristics

**Figure 2:**
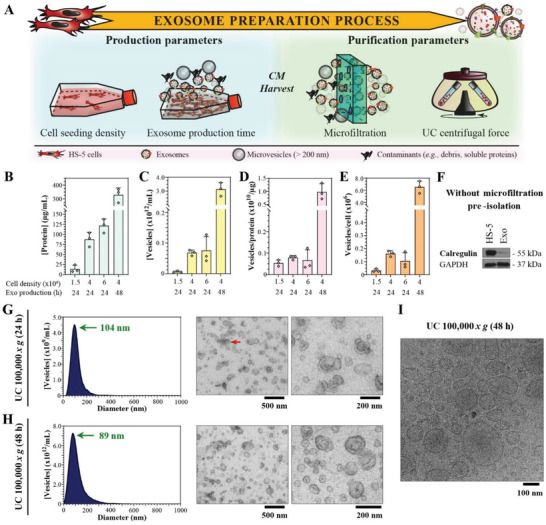
The preparation conditions impact the exosome yield and purity. A) Scheme illustrating the exosome preparation process. Investigated production and purification parameters are indicated. B) Protein concentration, C) vesicle concentration, D) vesicle‐to‐protein ratio, and E) vesicles per cell of HS‐5 exosomes isolated by UC at 100 000 × *g*. The *x*‐axis specifies the cell seeding density per 150 cm^2^ and exosome (Exo) production time. Data represent mean + SD, *n* = 3. F) Western blot analysis of exosomes purified by UC at 100 000 × *g* without microfiltration, for the contaminant calregulin. Exosomes were produced for G) 24 h or H) 48 h and isolated by UC at 100 000 × *g*. NTA size distributions (left panels) and TEM images (right panels) of exosomes. Green arrows mark the main vesicle population with their modal diameter and red arrows indicate vesicle aggregates. I) Cryo‐TEM image of exosomes produced over 48 h and isolated by UC at 100 000 × *g*. Experiments (panel (F); left panels in (G) and (H)) were performed in triplicates and a representative image/plot is shown.

Selected production and purification parameters were screened to maximize the yield of intact vesicles, while maintaining the purity of the exosome preparation (**Figure** **2**A).

The effects of the cell seeding density and exosome production time on the vesicle yield were first investigated. Importantly, the HS‐5 cell viability was greater than 95% for all tested conditions, hence ruling out the risk of excessive contamination with dead cells, debris or apoptotic bodies. Moreover, 6 × 10^6^ cells/150 cm^2^ was the maximal seeding density investigated as overconfluence was observed at higher plating densities. Vesicle yields increased as a function of the cell seeding density until it plateaued at a seeding density of 4 × 10^6^ cells/150 cm^2^, which corresponded to ≈80% cell confluence at the onset of the exosome production (Figure 2B,C). Purity of the exosomes produced from different cell numbers was similar, as indicated by the virtually stable vesicle‐to‐protein ratio (Figure 2D). In contrast, the normalized vesicle yield per cell was highest for a seeding density of 4 × 10^6^ cells/150 cm^2^ (Figure 2E), rendering it the optimal cell seeding density maintained in following experiments. Extending the exosome production to 48 h significantly improved the exosome yields compared to 24 h (Figure 2B,C,E). Moreover, the higher vesicle‐to‐protein ratio obtained after 48 h indicated a reduction of co‐isolated protein contaminants (Figure 2D). Similar trends were recently observed for primary amniotic fluid hMSC exosomes.^[^ ^33^ ^]^

Upon evaluation of pre‐isolation steps for their impact on the vesicle purity, microfiltration was demonstrated to be indispensable for warranting high vesicle purities, albeit it may diminish the vesicle yield.^[^ ^34^, ^35^ ^]^ This was indicated by the appearance of the contamination marker calregulin when differential low‐speed centrifugation on its own was used to process the conditioned medium (CM, i.e., cell culture supernatant after the exosome production period) (Figure 2F).

Excessive centrifugal forces may compromise the exosome morphology (i.e., aggregate formation).^[^ ^23^, ^36^ ^]^ While this was true for UC isolation at 150 000 × *g*, lower centrifugal forces of 100 000 × *g* resulted in an increased proportion of single non‐agglomerated vesicles with a smaller modal diameter of 104 ± 19 nm (Figure 2G; Figure S1C, Supporting Information). Furthermore, longer centrifugation times of up to 4 h have been reported to increase the vesicle yield without impairing their integrity.^[^ ^23^, ^37^ ^]^ While the vesicle recovery did not increase proportionally upon doubling the processing time from 70 to 130 min per UC run (1.2 ± 0.6 × 10^11^ vesicles mL^−1^ or 97 ± 11 µg protein mL^−1^ for 130 min per run vs 6.8 ± 1.0 × 10^10^ vesicles mL^−1^ or 87 ± 18 µg protein mL^−1^ for 70 min per run), the vesicle‐to‐protein ratio was slightly increased (1.2 ± 0.7 × 10^9^ vesicles µg^−1^ for 130 min per run vs 7.9 ± 0.1 × 10^8^ vesicles µg^−1^ for 70 min per run), indicating less co‐isolated contaminating proteins. Since these trends were not significant, a run time of 70 min was maintained to not overly prolong the purification process.

In summary, the optimal preparation conditions included a cell seeding density of 4 × 10^6^/150 cm^2^, a 48‐h vesicle production period, a microfiltration step, and a centrifugal force of 100 000 × *g* for 70 min per run. The resulting exosomes eventually had a modal diameter of 89 ± 7 nm and were not aggregated (Figure 2H; Figure S1D, Supporting Information). Importantly, the exosome enrichment and purity were verified by immunoblotting (Figure S2, Supporting Information) and the exosome functionality was corroborated by measuring their intrinsic CD73 activity (3.9 ± 0.6 U mg^−1^). Intact vesicle structures were confirmed by cryo‐TEM analysis (Figure 2I).

### 2.3 HS‐5 Exosome Characteristics Remain Stable upon Storage

The stability of HS‐5 exosomes was monitored after short‐ and long‐term storage at different temperatures. Incubation for 48 h at 37 °C did neither significantly alter the investigated physical properties nor the CD73 activity of the exosomes compared to 24 h and time zero, verifying that exosome properties remained consistent during the 48‐h vesicle production period (Figure S3A–D, Supporting Information). Upon long‐term storage (6 months) at −20 and −80 °C, no significant changes were observed in the vesicles’ modal diameter, zeta potential or CD73 enzymatic activity (Figure S3B–D, Supporting Information), pleading for a preserved exosome integrity. Vesicle counts seemed to increase after 6 months storage at −80 °C, but not −20 °C, for reasons that remain to be understood (Figure S3A, Supporting Information).

### 2.4 HS‐5 Exosome Protein Signature Is Similar between Passages #05 and #15

**Figure 3:**
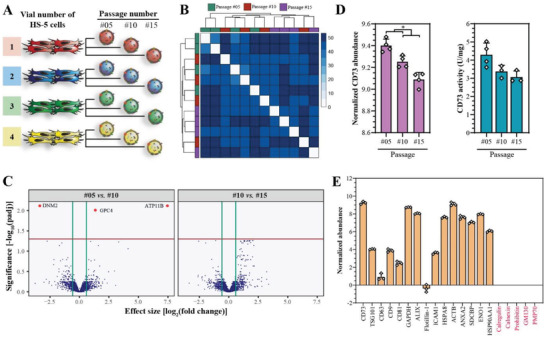
HS‐5 exosome protein profiles are consistent between cell passages #05 and #15. A) Four independent exosome batches each were produced from HS‐5 cells in passages #05, #10, and #15 and analyzed by quantitative proteomics. B) Heat map with hierarchical clustering of inter‐sample distances on the matrix of the overall normalized protein abundance. The color key is expressed in arbitrary units and darker blue colors indicate differences. *n* = 4 per passage number. C) Volcano plots of the quantitative protein analysis presented in two‐group comparisons (cell passage #05 vs #10 (left panel) and #10 vs #15 (right panel)). The *x*‐axis shows the effect size expressed as log2 fold change, which was calculated as ratio of the averaged normalized protein intensities of two passages (left: [average #10]/[average #05]; right: [average #15]/[average #10]). The *y*‐axis represents the significance expressed as negative decimal logarithm of the adjusted *p*‐value (−log10(padj)). The horizontal line indicates a false discovery rate (FDR) of 0.05, and the vertical lines indicate an absolute fold change of 1.5. *n* = 4 per passage number. D) Normalized CD73 abundance (left panel) and CD73 activity (right panel) are shown as a function of cell passages. Data represent mean + SD, *n* = 3–4. Significance was calculated with an ordinary two‐way ANOVA followed by a post‐hoc Tukey's multiple comparison test, *p < 0.05. E) Exosome marker proteins (black labels) as well as contamination marker proteins (red labels) are shown as averaged normalized protein abundance from passages #05, #10, and #15. Data represent mean + SD, *n* = 3 (single data points present the average of the relative abundance per passage number #05, #10, and #15).

The exosome composition was monitored as a function of cell passage to identify the window of passages within which consistent exosome properties could be expected. Based on the observed consistent HS‐5 growth kinetics, four independent exosome batch replicates were produced for passages #05, #10, and #15 of the HS‐5 cell culture (**Figure** **3**A), and were analyzed by quantitative proteomics. A total of 2286 proteins were identified, from which 1787 were present in exosomes from cells of all passages (Figure S4A, Supporting Information). Generally, all exosome batches had a similar global protein composition, whereby the intra‐passage similarity was higher than the inter‐passage one, demonstrating robustness of the exosome batches independently produced from the same HS‐5 cell passage (Figure 3B). Similarity was verified by two‐group differential protein abundance analysis and *p*‐value distributions comparing passages #05 vs #10 and #10 vs #15 (Figure 3C; Figure S4B, Supporting Information). Only three proteins (DNM2, ATP11B, and GPC4) (Table S1, Supporting Information) were differentially abundant to a significant extent between passages #05 vs #10, but not between #10 vs #15 (Figure 3C; Figure S4C, Supporting Information). No clear differences were found in the levels of the top eight abundant proteins at the different passages (Figure S4D, Supporting Information). Albeit a significant downward trend was observed in the CD73 protein levels with increasing passage number, it resulted in no significant reduction of the CD73 activity and was hence not considered as relevant (Figure 3D). Furthermore, in line with the immunoblot analysis, quantitative proteomics confirmed the presence of exosome markers as well as sample purity (Figure 3E).

Remarkably, a total number of 144 proteins with a documented role in wound healing were identified in the HS‐5‐derived exosomes (Figure S4E, Supporting Information). These proteins could be classified by their function in extracellular matrix organization, proliferation, migration, epithelialization, collagen and glycosaminoglycan biosynthesis, and angiogenesis (Figure S4E and Table S2, Supporting Information). Importantly, protein levels were similar intra‐ and inter‐passage (Figure S4F, Supporting Information), suggesting that no significant differences in the biological activity of the exosomes produced from different HS‐5 cell passages should be expected.

Collectively, exosome protein signatures were shown to be constant between passages #05 and #15, indicating that the vesicles’ biological profiles can be considered consistent within this passage range. Moreover, several well‐known proteins involved in processes required for wound healing, including extracellular matrix proteins and their integrin receptors or growth factors and their receptors,^[^ ^38^ ^]^ were detected in HS‐5‐derived exosomes, verifying their potential function in wound healing.

### 2.5 HS‐5 Exosomes Have an In Vitro Wound Healing and Angiogenic Potential, Which Is Partially Driven by CD73 and Lipids

Primary hMSC exosomes have been previously emphasized to promote wound healing, tracing their activity to their protein and nucleic acid content in the first place.^[^ ^10^, ^39^, ^40^, ^41^ ^]^ Here, the involvement of exosomal lipids and the abundantly present enzyme CD73 in HS‐5 exosome‐driven activities relevant for cutaneous wound healing was studied using in vitro scratch and angiogenesis assays on relevant cell culture models (i.e., keratinocytes, fibroblasts, and endothelial cells). Based on the lipidomic characterization of HS‐5 exosomes (Figure S5, Supporting Information), synthetic exosome‐like liposomes (SELL) were designed to assess the activity of the most abundant HS‐5 exosomal lipids.

**Figure 4:**
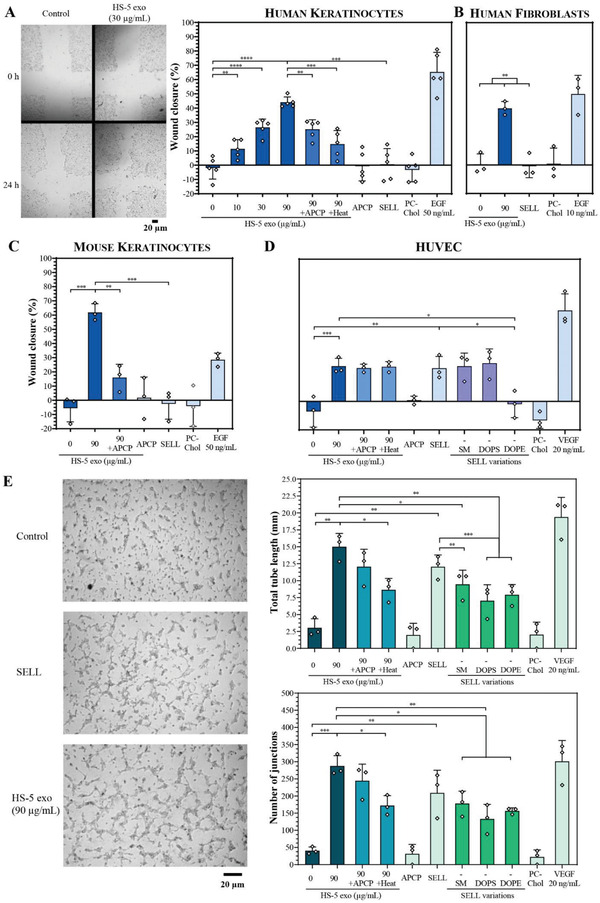
Purified HS‐5 exosomes promote scratch wound closure and endothelial cell tube formation in vitro. Scratch wound healing assay using A) human HaCaT keratinocytes with representative bright‐field images, B) primary human fibroblasts, C) immortalized mouse keratinocytes, and D) HUVEC. Cells were treated with HS‐5 exosomes (0, 10, 30, and 90 µg protein mL^−1^), CD73‐inhibited HS‐5 exosomes (90 µg protein mL^−1^, 30 × 10^−6^ m APCP), heat‐inactivated HS‐5 exosomes (90 µg protein mL^−1^, 2.5 h, 65 °C), SELL (1.3 × 10^11^ vesicles mL^−1^) or variations thereof (either without SM, or DOPS or DOPE), PC‐Chol liposomes (1.3 × 10^11^ vesicles mL^−1^), APCP (30 × 10^−6^ m), or buffer control. Epidermal growth factor (EGF, 10 or 50 ng mL^−1^) or vascular endothelial growth factor (VEGF, 20 ng mL^−1^) were used as positive controls. The wound closure at 24 h after scratch wounding was normalized to the medium control. E) Capillary tube formation assay in HUVEC. Representative bright‐field images and quantification of the total tube length and the number of junctions. HUVEC were treated with HS‐5 exosomes (90 µg protein mL^−1^), CD73‐inhibited HS‐5 exosomes (90 µg protein mL^−1^, 30 × 10^−6^ m APCP), heat‐inactivated HS‐5 exosomes (90 µg protein mL^−1^, 2.5 h, 65 °C), SELL (1.3 × 10^11^ vesicles mL^−1^) or variations thereof, PC‐Chol liposomes (1.3 × 10^11^ vesicles mL^−1^), APCP (30 × 10^−6^ m), or buffer control or the positive control VEGF (20 ng mL^−1^), and analyzed 20 h after treatment. A–E) Data represent mean + SD, *n* = 3–5. Significance was calculated with an ordinary two‐way ANOVA followed by a post‐hoc Tukey's multiple comparison test, *p < 0.05, ^**^ *p* < 0.01, ^***^ *p* < 0.001, ^****^ *p* < 0.0001.

HS‐5‐derived exosomes significantly and dose‐dependently promoted the scratch wound closure of immortalized, but non‐tumorigenic human keratinocytes (HaCaT cells^[^ ^42^ ^]^) compared to the buffer control (**Figure** **4**A). Depleting the CD73 activity by either thermal treatment of the HS‐5 exosomes or simultaneous stimulation with the CD73 inhibitor adenosine 5′‐(*α*,*β*‐methylene)diphosphate (APCP) partially reduced the exosomes’ wound closure‐promoting activity (Figure 4A; Table S3, Supporting Information). This confirmed the involvement of proteins in this process, and suggested that thermostable components other than CD73 (e.g., lipids, nucleic acids) contributed as well. On the other hand, SELL had no clear effect on the wound closure rate (Figure 4A). Similarly, liposomes formulated with 1,2‐dioleoyl‐*sn*‐glycero‐3‐phosphocholine (DOPC) and cholesterol (PC‐Chol), which are commonly used for drug delivery purposes,^[^ ^43^, ^44^ ^]^ were inactive. In the presence of the proliferation inhibitor mitomycin C, the HS‐5 exosomes’ effects were less pronounced (Figure S6, Supporting Information), indicating a predominant pro‐mitogenic and a less marked pro‐migratory activity under the tested experimental conditions. These findings revealed that CD73 and exosome components other than lipids expedited the scratch wound closure in the HaCaT cell model.

HS‐5 exosomes further promoted scratch wound closure of primary human fibroblasts and immortalized mouse keratinocytes, while liposomes irrespective of their composition were inactive (Figure 4B,C). Moreover, CD73 inhibition partially abrogated the exosomes’ effect on the scratch wound closure of immortalized mouse keratinocytes, reaffirming its pivotal role in this process (Figure 4C; Table S3, Supporting Information). HS‐5 exosomes also promoted scratch wound closure of primary human umbilical vein endothelial cells (HUVECs), although to a lesser extent compared to their effect on fibroblasts and keratinocytes (Figure 4D). Intriguingly, SELL, which were inactive on fibroblasts and keratinocytes, had a similar effect on the wound closure rate in HUVEC as HS‐5 exosomes, suggesting that specific exosome lipids contained in the liposome formulation were essential contributors to the HS‐5 exosome efficacy, at least in the tested concentration. Confirming that mainly lipids promoted the exosomes’ activity, neither thermal treatment nor CD73 inhibition showed any detectable effects on the exosome activity on HUVEC (Figure 4D). A closer investigation of the lipid species contained in the SELL formulation revealed that 1,2‐dioleoyl‐*sn*‐glycero‐3‐phosphoethanolamine (DOPE), but neither sphingomyelin (SM) nor 1,2‐dioleoyl‐*sn*‐glycero‐3‐phospho‐l‐serine (DOPS), significantly contributed to the observed effects, as indicated by the SELL’ loss‐of‐activity upon depletion of DOPE (Figure 4D). On the other hand, PC‐Chol liposomes were inactive. Notably, the modal diameters of SELL and its variations as well as PC‐Chol liposomes were similar (Table S4, Supporting Information), also to HS‐5 exosomes, hence ruling out potential effects originating from differences in the size.^[^ ^45^ ^]^

Angiogenesis plays a pivotal role in wound healing and primary hMSC‐derived exosomes have a reported pro‐angiogenic capability.^[^ ^38^, ^46^ ^]^ Therefore, the activity of the HS‐5‐derived exosomes was investigated in an in vitro capillary tube formation assay using HUVEC. Compared to the control, HS‐5 exosomes significantly increased the total tube length, which reflects the tube formation, and the number of junctions, which represents the complexity of the tube network (Figure 4E). Consistent with the findings from the scratch wound healing assay with HUVEC, SELL were almost as efficient as the exosomes in the tested concentration. All three lipid species (i.e., DOPS, DOPE, and SM) seemed to be involved in the SELL’ angiogenic activity as seen by a significant reduction of the total tube length and number of junctions following either DOPS or DOPE or SM depletion compared to HS‐5 exosomes (Figure 4E). Effects seemed to be even stronger on the total length of the capillary tube network as here, a significant difference was also seen compared to SELL. PC‐Chol liposomes were again inactive. Moreover, CD73 inhibition resulted in a non‐significant reduction of the HS‐5 exosome activity. Thermal treatment, which inactivates thermolabile exosomal proteins including CD73 (Table S3, Supporting Information), significantly diminished the exosome effects to levels approaching those of the exosomal lipids (Figure 4E).

Taken together, these results suggested that HS‐5 exosomes directly promote the scratch wound closure of keratinocytes, fibroblasts, and endothelial cells, at least partially via CD73 activity. Moreover, HS‐5 exosomes showed a direct pro‐angiogenic activity, which was mainly dependent on exosomal lipids.

### 2.6 HS‐5 Exosomes Show a Promising Therapeutic Potential for Cutaneous Wound Healing in Healthy Mice

**Figure 5:**
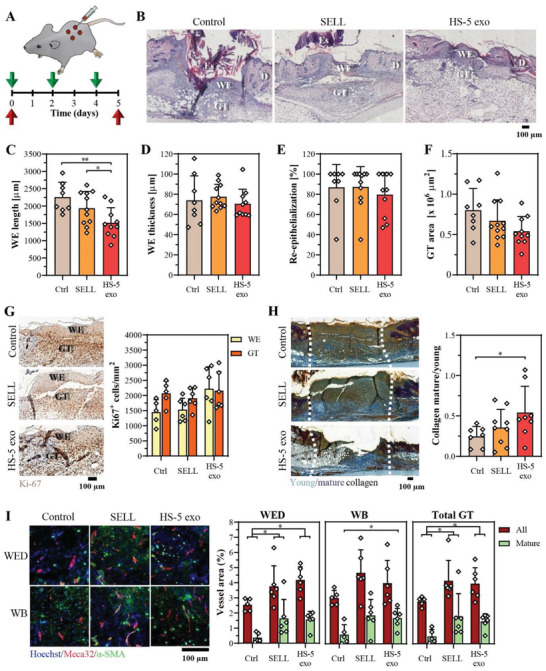
HS‐5 exosomes in a dosage of 15 µg are partially effective in skin wound healing. A) Full‐thickness excisional wounds in female C57BL/6 mice were treated with HS‐5 exosomes (1.5 × 10^11^ vesicles or 15 µg protein), SELL (1.5 × 10^11^ vesicles), or vehicle control. Green arrows indicate the day of intradermal injection, and red arrows indicate wounding and sacrificing of mice. B–F) Histomorphometric analysis of wounds at day 5 after injury. B) Representative photomicrographs of H&E‐stained wound sections (Es = eschar; WE = wound epidermis/epithelium; D = dermis; GT = granulation tissue), C) average epithelium length and D) thickness, E) percentage of re‐epithelialization, and F) GT area. G) Representative wound sections with Ki‐67 staining of proliferating cells and quantification thereof in the WE and GT. H) Representative Herovici‐stained wound sections and quantification of the mature‐to‐young collagen ratio. White dashed lines outline the wound area. Outliers were identified by the ROUT method (*Q* = 1%) and removed (one outlier per treatment and control group). I) Blood vessel formation and maturation in 5‐day wounds. Vessel area is expressed as the percentage of the total area of the WED, WB, and total GT (GT = WED + WB). Representative wound sections co‐stained with Meca32 (red) and *α*‐SMA (green). Mature vessels, which are surrounded by vascular smooth muscle cells, show co‐staining of both markers. Cell nuclei were counterstained with Hoechst 33342 (blue). C–I) Data represent mean + SD, *n* = 6–11 wounds from 6 mice per treatment group. Significance was calculated with an unpaired, non‐parametric Mann–Whitney *U* test, *p < 0.05, ^**^ *p* < 0.01.

Since HS‐5 exosomes showed a positive effect in cell‐based wound closure and angiogenesis assays, their efficacy was further investigated in a pilot full‐thickness excisional wound healing study in healthy mice. Exosomes were compared to SELL to distinguish between biological activity stemming from either lipids or proteins/nucleic acids carried by the exosomes. HS‐5 exosomes (15 µg protein or 1.5 × 10^11^ vesicles), SELL (1.5 × 10^11^ vesicles), or vehicle control were injected intradermally at the wound edges immediately post‐wounding and then at day 2 and day 4 (**Figure** **5**A). Wounds were analyzed at day 5 after injury, a time point when re‐epithelialization and granulation tissue formation are clearly visible.^[^ ^47^, ^48^ ^]^

Wound healing in healthy mice is highly optimized and therefore, a significant enhancement of the overall healing process is rarely observed.^[^ ^48^ ^]^ Indeed, histomorphometric analysis of hematoxylin/eosin (H&E)‐stained wound sections at day 5 showed similar wound closure rates in the different treatment groups (Figure 5B; Figure S7A, Supporting Information). While the wound epithelium length, which reflects the migration distance, even decreased following either exosome or SELL treatment compared to the control, no clear differences in the thickness of the wound epidermis, re‐epithelialization and wound contraction were observed (Figure 5C–E; Figure S7B, Supporting Information). These observations were confirmed by semi‐quantitative wound scoring (Figure S7C,D, Supporting Information). There was a non‐significant trend towards a reduced granulation tissue area in either exosome‐ or SELL‐treated wounds (Figure 5F). Ki67‐staining of proliferating cells showed a mild, but non‐significant increase in cellular proliferation in the wound epidermis following HS‐5 exosome vs control treatment, while no effect was seen after treatment with SELL (Figure 5G). Neither exosomes nor SELL clearly enhanced the cell proliferation rate in the granulation tissue. Herovici staining, which distinguishes between young and mature collagen fibers, showed an increase in the ratio of mature to young collagen fibers within the granulation tissue in exosome‐ and SELL‐treated wounds (Figure 5H; Figure S7E, Supporting Information), suggesting a potential function of exosomes in collagen maturation during the wound healing process. Most importantly, both HS‐5 exosomes and SELL equally augmented the total area covered by blood vessels in the granulation tissue (i.e., area that stained positive for the vascular endothelial cell marker Meca32) compared to the vehicle control (Figure 5I). Interestingly, both the area and mean blood vessel sizes were only significantly increased at the wound edge, suggesting a potential local action close to the injection sites (Figure 5I; Figure S7F, Supporting Information). Similar trends were observed for vascular maturation (i.e., blood vessels surrounded by *α*‐smooth muscle action (*α*‐SMA)‐expressing vascular smooth muscle cells) (Figure 5I; Figure S7F, Supporting Information). The area covered by *α*‐SMA positive cells outside of vessels (i.e., myofibroblasts) did not reveal significant differences in the different treatment groups (Figure S7G, Supporting Information).

## 3 Discussion

Apart from few exceptions,^[^ ^37^, ^49^ ^]^ studies investigating currently available exosome isolation methods provide only insufficient information on the impact of specific production and/or purification parameters on the vesicles’ characteristics. Moreover, in most cases the exosome formulation quality assessment is limited to the minimally required exosome identification characteristics (i.e., size, concentration, purity, identity, and morphology).^[^ ^23^, ^30^, ^31^ ^]^ In this work, the optimal conditions for the production and purification of bioactive HS‐5 exosomes were systematically investigated, leading to the development of a standardized method that reproducibly yielded functional exosomes with high efficiency and purity. In addition, a rapid and cell‐free enzymatic assay was introduced to monitor the exosome functionality, and indirectly the vesicle integrity. Quantitative proteomics allowed to determine the cell passage range delivering exosomes with a consistent global protein composition.

Good purification efficiencies have been reported for UF and SEC, either individually or combined.^[^ ^26^, ^27^, ^28^ ^]^ However, the head‐to‐head comparative analysis of UC and UF‐SEC clearly identified UC as the superior exosome purification approach in terms of yield, purity, and enzymatic functionality. It is well known that EV can non‐specifically bind to and/or block the UF membranes, potentially leading to an increased rejection of subpore‐sized contaminants and EV loss.^[^ ^6^, ^50^ ^]^ Moreover, the active shear forces may provoke the formation of vesicle and/or contaminating protein clusters and can even damage the vesicles, including their membrane enzymes.^[^ ^6^ ^]^ This is particularly problematic in view of the filtration of larger volumes, such as the one used in this work. The volume of clarified CM (CCM) was more than twice the one used by others^[^ ^26^, ^27^, ^28^ ^]^ and could therefore explain the overall poor UF‐SEC performance. This CCM volume was in fact required to produce a sufficient exosome quantity for downstream analyses, and to determine the vesicle recovery after both isolation methods based on comparable volumes. The 70 nm pore‐sized column used for SEC, a pore size also employed in previous studies,^[^ ^26^, ^51^, ^52^ ^]^ could present another possible source for EV loss, as only EV larger than the pore size can be efficiently separated from smaller contaminating proteins.^[^ ^51^ ^]^

During process optimization, it was found that the cultivation conditions and UC centrifugal force were key for a high yield and morphological integrity of the exosome formulation. Furthermore, only differential UC in combination with microfiltration could purify the vesicles efficiently from contaminating proteins. Moreover, the global protein expression profile of HS‐5 exosomes was consistent between passages #05 and #15, which would also indicate stable vesicle properties during the production. Finally, physical properties and CD73 activity of HS‐5 exosomes remained generally unchanged upon long‐term storage at −20 and −80 °C, demonstrating good shelf life of the isolated vesicles, as was previously proposed.^[^ ^53^ ^]^

Exploiting the optimized and standardized isolation method, the role of selected exosome components involved in HS‐5 exosome‐promoted parameters relevant for cutaneous wound healing was subsequently investigated.

Quantitative proteomics verified that proteins involved in processes required for wound healing were abundantly present in HS‐5‐derived exosomes, among which the transmembrane enzyme CD73 was not reported yet. CD73, whose beneficial action in graft‐versus‐host‐disease^[^ ^12^ ^]^ and cartilage repair^[^ ^13^ ^]^ was previously emphasized, was found to play a fundamental role in the exosome‐mediated acceleration of the scratch wound closure of keratinocytes. The loss of activity that was observed upon heating the HS‐5 exosomes at 65 °C also suggests that thermal treatment at higher temperatures, which was previously shown to not substantially affect the morphology or uptake behavior of the vesicles,^[^ ^54^ ^]^ should be carefully examined as it may be detrimental to the exosome functionality. Notably, the HaCaT cell model used in this work is frequently used to study skin biology for it retains a normal differentiation capacity, though differing in several aspects from primary human keratinocytes.^[^ ^42^ ^]^

In endothelial cells, exosomes and SELL showed comparable effects on both the scratch wound closure and capillary tube formation. Sphingolipids and glycerophospholipids, which were the main components of the SELL and were as well abundantly present in the HS‐5 exosomes, were previously shown to promote capillary blood vessel network formation and maturation as well as proliferation/migration of human endothelial cells.^[^ ^55^, ^56^ ^]^ Here, a leading role of DOPE in promoting the scratch wound closure and, together with DOPS and SM, tube formation activity was shown. The fact that thermal inactivation and/or CD73 inhibition not or only minimally affected the HS‐5 exosomes’ activity on endothelial cells compared to keratinocytes indicated the strong interplay between the exosomal component activity and the target cell type.

In a pilot experiment performed with healthy mice, HS‐5 exosomes as well as SELL had no clear effects on the wound closure, wound epidermis thickness, re‐epithelialization, or wound contraction. This was not unexpected, since the wound healing process in healthy mice is highly optimized.^[^ ^38^, ^48^ ^]^ Surprisingly, however, both treatments even reduced the wound epidermis length and granulation tissue area, but for reasons that remain unclear. In contrast, HS‐5 exosomes but not their liposomal counterparts, slightly increased the cell proliferation rate at the wound site in healthy mice, suggesting that the dosage applied in vivo might need to be increased to produce significant effects. Most importantly, both HS‐5 exosomes and SELL comparably promoted angiogenesis, in agreement with the in vitro results. Effects were stronger close to the injection sites, suggesting either a mostly local activity of the exosomes and/or liposomes, or an enhancement of the natural healing process by extending the vascular network from the wound edge towards the wound bed.^[^ ^57^ ^]^ Furthermore, exosomes and SELL favored an increased production of mature collagen fibers, which is expected to result in an early regain of tensile strength.^[^ ^58^ ^]^ It remains to be determined whether the exosomes and/or SELL also affect the scarring response and can even limit this effect, as was previously suggested for exosomes produced from adipose‐derived hMSC.^[^ ^59^ ^]^

Taken together, these encouraging results suggest that lipids may play an important part in the wound healing activity of exosomes, warranting further investigations to more clearly define the dosage at which their effect predominates. The in vivo effects of other exosome components (e.g., CD73 or other proteins with a previously reported wound healing promoting activity) may become apparent at higher injected doses, which were not tested in this study. Moreover, the dosing regimen could also affect the therapeutic outcome. It is believed that exosomes are rapidly cleared from the injection site via the lymphatic system,^[^ ^60^ ^]^ emphasizing that a more frequent injection or the use of a sustainable release formulation and/or topical application of the exosomes and subsequent coverage with a dressing could be beneficial.^[^ ^9^, ^61^ ^]^ It was indeed recently demonstrated that a sustained exosome release in the wound bed via a hydrogel formulation was more effective than a single‐dose application, likely due to a steady‐state exosome concentration at the wound site.^[^ ^9^ ^]^ Finally, an animal model for impaired wound healing (e.g., diabetic wounds,^[^ ^9^, ^48^ ^]^ or use of skin burn injury^[^ ^10^ ^]^) might reveal additional and/or more potent effects of exosomes and SELL, as they directly intervened in dysregulated healing processes.^[^ ^15^, ^46^ ^]^ Noteworthily, the results in this work have been obtained with exosomes derived from the HPV‐16 E6/E7 transformed human bone marrow stromal cell line HS‐5, which allowed to obtain large numbers of exosomes with consistent composition and activity. In the future, it will be important to revalidate the results with primary hMSC as they better mimic a physiological cell behavior.

In conclusion, the bioactivity of HS‐5 exosomes appeared to be mediated by a broader range of biomacromolecules beyond yet reported proteins and nucleic acids. In particular, the transmembrane enzyme CD73 and exosomal lipids were shown to play an important role in the regulation of processes relevant for wound healing by HS‐5‐derived exosomes, providing a solid gateway for further in‐detail investigations of the underlying molecular mechanisms these components trigger in target cells/tissue. Additionally, the key conditions of the exosome preparation process that critically determine the exosome quality were established, demonstrating the strong interdependence of the preparation conditions and the exosome formulation quality.

## 4 Experimental Section

##### Materials

The HPV‐16 E6/E7 transformed human bone marrow stromal cell line HS‐5 was purchased from ATCC (Manassas, VA, USA). Primary human dermal fibroblasts and immortalized human keratinocytes (HaCaT cell line) were kindly provided by Dr. Hans‐Dietmar Beer (Department of Dermatology, University Hospital Zurich, Zurich, Switzerland) and Dr. P. Boukamp (Leibniz Institute for Environmental Medicine, Düsseldorf, Germany), respectively. Mouse keratinocytes were isolated as described previously.^[^ ^62^ ^]^ Primary HUVEC were obtained from ScienCell Research Laboratories (Carlsbad, CA, USA). Defined keratinocyte SFM, Dulbecco's Modified Eagle's Medium (DMEM), EGF, VEGF, fetal bovine serum (FBS), GlutaMAX, l‐glutamine, penicillin, streptomycin, HEPES 1 m solution, and phosphate buffered saline (PBS) were obtained from Thermo Fisher Scientific (Waltham, MA, USA). Endothelial basal medium (EBM) was bought from Lonza Group AG (Basel, Switzerland). APCP, Minimum Essential Medium Eagle (MEM) Spinner modification, cholera toxin, ethanolamine, hydrocortisone, insulin, *o*‐phosphorylethanolamine, and transferrin were purchased from Sigma‐Aldrich (St. Louis, MO, USA). Sodium chloride (NaCl), sodium fluoride (NaF), sodium dodecyl sulfate (SDS), sodium orthovanadate (Na_3_VO_4_), Tris, sodium citrate, skim milk powder, bovine serum albumin (BSA), *β*‐mercaptoethanol, Triton X‐100 and polysorbate 20, chloroform ReagentPlus, glutaraldehyde, paraformaldehyde, paraffin, methylcellulose, uranyl acetate, xylene, hematoxylin, eosin, Hoechst 33342 as well as SM (egg, chicken), and cholesterol were also purchased from Sigma‐Aldrich. PureCol bovine collagen solution (3 mg mL^−1^) was obtained from Advanced BioMatrix (Carlsbad, CA, USA). Ethylenediaminetetraacetic acid (EDTA), acetone, and acetic acid were obtained from Fluka Chemie AG (Buchs, Switzerland). cOmplete EDTA‐free Protease Inhibitor Cocktail was from F. Hoffmann‐La Roche Ltd. (Basel, Switzerland). Bromophenol blue was purchased from Alfa Aesar (Haverhill, MA, USA). Adenosine‐5′‐monophosphate (AMP), glycerol, and phenylmethylsulfonyl fluoride (PMSF) were obtained from Acros Organics (Geel, Belgium). Malachite green was bought from Bender & Hobein GmbH (Munich, Germany). Ammonium molybdate tetrahydrate was from abcr GmbH (Karlsruhe, Germany). Calcium chloride (CaCl_2_), ethanol, 10 kDa molecular weight cut‐off (MWCO) Amicon Ultra‐0.5 mL Centrifugal Filters, and 100 kDa MWCO Centricon Plus‐70 Centrifugal Filter Units were obtained from Merck KGaA (Darmstadt, Germany). The lipids DOPC, DOPS, and DOPE (all >99% purity) were obtained from Avanti Polar Lipids (Alabaster, AL, USA). Primary mouse monoclonal antibodies anti‐CD73 (sc‐32299), anti‐CD63 (sc‐5275), anti‐CD9 (sc‐13118), anti‐TSG101 (sc‐7964), anti‐calregulin (sc‐373863), and anti‐GAPDH (sc‐47724) as well as the Western Blotting Luminol Reagent were purchased from Santa Cruz Biotechnology Inc. (Dallas, TX, USA). The secondary HRP‐conjugated polyclonal goat anti‐mouse immunoglobulin was obtained from Dako Denmark A/S (Glostrup, Denmark). Purified rat anti‐mouse pan‐endothelial cell antigen (Meca32) was obtained from BD Biosciences (Franklin Lakes, NJ, USA), mouse monoclonal anti‐actin *α*‐smooth muscle‐FITC antibody from Sigma‐Aldrich. Secondary biotinylated anti‐rabbit IgG and secondary Cy3 AffiniPure donkey anti‐mouse IgG antibody were from Jackson ImmunoResearch Europe Ltd. (West Grove, PA, USA). Primary rabbit monoclonal anti‐Ki67 (ab15580) antibody was obtained from Abcam (Cambridge, UK). qEVoriginal/70 nm SEC columns were purchased from Izon Science Ltd. (Christchurch, New Zealand). Immun‐Blot PVDF membranes with a pore size of 0.2 µm were from Bio‐Rad Laboratories Inc. (Hercules, CA, USA). Fuji medical X‐ray films were purchased from FUJIFILM Europe GmbH (Düsseldorf, Germany). The 100 nm pore‐sized polycarbonate filter membranes were bought from Sterlitech Corporation (Kent, WA, USA). Corning 96‐well Clear Polystyrene Microplates were purchased from Corning Inc. (Corning, NY, USA). Tissue culture test plates with 48 wells and sterile 0.22 µm pore‐sized filters were obtained from TPP Techno Plastic Products AG (Trasadingen, Switzerland). Carbon‐coated grids and 300‐mesh lacey carbon‐coated copper grids were from Quantifoil Micro Tools GmbH (Grosslöbichau, Germany). Reversed phase nanoEase M/Z Symmetry C18 Trap Column (100 Å, 5 µm, 180 µm × 20 mm) and nanoEase M/Z C18 HSS T3 Column (100 Å, 1.8 µm, 75 µm × 250 mm) as well as Acquity UPLC HSS T3 Column (1.8 µm, 150 µm × 50 mm) were obtained from Waters Corporation (Milford, MA, USA). Tissue‐freezing medium was obtained from Leica Biosystems (Wetzlar, Germany).

##### Cell Culture

HS‐5 cells, HaCaT, and human fibroblasts were cultivated in DMEM supplemented with 100 U mL^−1^ penicillin, 100 µg mL^−1^ streptomycin, and 10% v/v FBS. Mouse keratinocytes were maintained in Defined Keratinocyte SFM and MEM in a ratio 2:1 v/v supplemented with 1.7 µg mL^−1^ insulin, 3.3 µg mL^−1^ transferrin, 0.5 µg mL^−1^ *o*‐phosphorylethanolamine, 0.2 µg mL^−1^ ethanolamine, 0.1 µg mL^−1^ hydrocortisone, 15 × 10^−6^ m CaCl_2_, 0.7 × 10^−3^ m GlutaMAX, 100 U mL^−1^ penicillin, 100 µg mL^−1^ streptomycin, 67 × 10^−12^ m cholera toxin, 2.7% v/v chelated FBS, and 10 ng mL^−1^ EGF. HUVEC were cultured in EBM containing 100 U mL^−1^ penicillin, 100 µg mL^−1^ streptomycin, 20% v/v FBS, 2 × 10^−3^ m l‐glutamine, and 10 µg mL^−1^ hydrocortisone. All cells were cultured under humidified conditions at 37 °C and 5% CO_2_ in a Heracell 240i CO_2_ Incubator (Thermo Fisher Scientific) and routinely tested to be negative for mycoplasma using the MycoAlert Mycoplasma Detection kit according to the manufacturer's instructions (Lonza Group AG). Cell viability was determined by the trypan blue exclusion assay as described previously.^[^ ^63^ ^]^

##### Production of HS‐5‐Derived Exosomes

Exosomes were produced according to a previously published protocol,^[^ ^23^ ^]^ which was customized. In brief, 4 × 10^6^ HS‐5 cells were seeded in a volume of 15 mL complete growth medium per T150 cell culture flask and cultured for 40 h. For exosome production, cells were washed twice with 6 mL PBS and further cultured in 18 mL fresh, serum‐free medium for 24 and 48 h, respectively. The CM was harvested and centrifuged at 4 °C and 2000 × *g* for 5 min, immediately followed by 10 000 × *g* for 15 min using a Sorvall RC 6 PLUS centrifuge equipped with a SA‐600 Fixed Angle Rotor (Thermo Fisher Scientific). The supernatant was filtered through a 0.22 µm pore‐sized filter to obtain the CCM. The CCM was stored at −20 °C.

##### UC

UC was performed as described previously.^[^ ^23^ ^]^ A volume of 396 mL CCM was centrifuged at 4 °C and either 100 000 × *g* or 150 000 × *g* for 70 min using an Optima XE‐90 ultracentrifuge equipped with a Type 45 Ti Fixed‐Angle Titanium Rotor (Beckman Coulter Life Sciences, Indianapolis, IN, USA). Exosome‐containing pellets were resuspended in 10 mL PBS, pooled, and subjected to a second ultracentrifugation round under equal conditions. The purified exosome pellet was resuspended in 200 µL PBS and stored at −20 °C. In case of CD73 activity determination, isolated exosomes were resuspended in HEPES buffered saline (HEBS, 20 × 10^−3^ m HEPES, 150 × 10^−3^ m NaCl, pH 7.4).

##### UF‐SEC

UF‐SEC was carried out as described before.^[^ ^26^ ^]^ A volume of 360 mL CCM was concentrated to ≈400 µL by centrifugation at 4 °C and 3500 × *g* using 100 kDa MWCO Centricon Plus‐70 Centrifugal Filter Units. The concentrated CCM was applied on qEVoriginal/70 nm SEC columns and 500 µL fractions were collected. Due to the low exosome amount present per fraction, exosome identification guidelines according the International Society for Extracellular Vesicles^[^ ^30^, ^31^ ^]^ could not be followed. Alternatively, exosome‐containing fractions were identified by a protein/CD73 activity cut‐off requiring only very low amounts of exosomal protein. Their protein concentration was determined by the Micro bicinchoninic acid (BCA) assay (see section “Protein Quantification”). To determine the CD73 enzymatic activity of the fractions, 3 µL per fraction was incubated with 40 × 10^−6^ m AMP in a reaction volume of 60 µL for 20 h at room temperature (RT). AMP hydrolysis was then determined by a malachite green assay (see section “CD73 Enzymatic Activity Assay”). Fractions with less than 50 µg mL^−1^ protein and more than 50% AMP hydrolysis (qEV1/qEV2: fractions 8–13; qEV3: fractions 7–14) were concentrated by centrifugation at 4 °C and 14 000 × *g* in 10 kDa MWCO Amicon Ultra‐0.5 mL Centrifugal Filters to a final volume of ≈120 µL.

##### Protein Quantification

The total protein amount of the exosomes was determined by the Micro BCA assay according to the manufacturer's instructions (Thermo Fisher Scientific). Briefly, 2 µL exosomes were diluted with nanopure water to a volume of 150 µL and added per well of a Corning 96‐well Clear Polystyrene Microplate. After addition of 150 µL Micro BCA reagent per well, the solutions were incubated at 37 °C for 2 h and the absorbance at 562 nm was measured using a Tecan Infinite M200 (Tecan Group Ltd., Männedorf, Switzerland).

##### CD73 Enzymatic Activity Assay

To determine the CD73 activity of the HS‐5 exosomes, a previously published malachite green assay was adapted.^[^ ^64^ ^]^ Briefly, 60 µL containing 0.1 µg exosome protein and 24 µmol AMP were incubated in a Corning 96‐well Clear Polystyrene Microplate for 10 min at RT. To stop the enzymatic reaction, 40 µL color reagent (0.034% w/v malachite green, 1.55% w/v ammonium molybdate tetrahydrate, 0.0625% v/v polysorbate 20) was added per well and incubated for 1 h at RT. The absorbance was measured at 620 nm using a Tecan Infinite M200.

##### Liposome Preparation

Liposomes were prepared by the thin‐film hydration method.^[^ ^65^ ^]^ For PC‐Chol liposomes, DOPC and cholesterol were dissolved in chloroform ReagentPlus at a molar ratio of 70:30. For SELL, a mixture composed of DOPC, DOPS, DOPE, SM, and cholesterol was dissolved in chloroform ReagentPlus at a molar ratio of 21:14:17.5:17.5:30.^[^ ^43^, ^66^ ^]^ As indicated in the results section, variations of SELL were created by replacing either DOPS, DOPE, or SM by DOPC. Thin lipid films were generated by initial solvent removal at 2000 Pa with a rotary evaporator (Büchi Labortechnik AG, Flawil, Switzerland) and then dried in vacuo for over 12 h at RT. The thin lipid film was then rehydrated in PBS at 55 °C to a final total lipid concentration of 10 × 10^−3^ m. Liposomes were immediately subjected to ten freeze–thawing cycles and extruded through two‐stacked 100 nm pore‐sized polycarbonate filter membranes at 55 °C. Liposome suspensions were stored at 4 °C.

##### NTA

The size profile and concentration of exosomes isolated from similar CCM volumes (see sections “UC” and “UF‐SEC”) were analyzed on a NanoSight NS300 equipped with a CMOS camera and a 488 nm laser source (Malvern Panalytical GmbH, Kassel, Germany) as well as on a ZetaView equipped with a CMOS camera and a 405 nm laser source (Particle Metrix GmbH, Meerbusch, Germany). The ZetaView was additionally used to determine the zeta potential of the vesicles as well as the concentration and diameter of the liposomes. Samples were diluted in PBS (1:100–1:1000 for exosomes produced over 24 h and 1:1 000 000 for exosomes produced over 48 h and for liposomes) to a concentration of 10^7^–10^8^ vesicles mL^−1^, giving 50–200 vesicles per frame. For the NanoSight, a video of 60 s was recorded, with the camera level set to 14, the slider shutter to 1259, and the slider gain to 366. Data was analyzed with the NanoSight NTA 3.1 Build 3.1.54 software setting a detection threshold of 4. For the ZetaView, the sensitivity was set to 85, the shutter to 150, and the frame rate to 30. Data was analyzed with the ZetaView software version 8.04.04 SP2 applying a bin class width of 5 nm, minimum brightness of 25, minimum area of 5, maximum area of 1000, and trace length of 15.

##### Western Blot

Western blot analysis was performed as described previously.^[^ ^67^, ^68^ ^]^ Briefly, 10^6^ HS‐5 cells were harvested, centrifuged at 300 × *g* for 5 min, washed with PBS and resuspended in 100 µL 1× lysis buffer (20 × 10^−3^ m Tris, 150  × 10^−3^ m NaCl, 5 × 10^−3^ m EDTA, 1% v/v Triton X‐100, 25 × 10^−3^ m NaF, 1 × 10^−3^ m PMSF, 1 × 10^−3^ m Na_3_VO_4_, cOmplete EDTA‐free Protease Inhibitor Cocktail). Cells were lysed on ice for 30 min and subsequently centrifuged at 4 °C and 10 000 × *g* for 10 min to collect the supernatant. Isolated exosomes were diluted 5:1 v/v in 5× lysis buffer and incubated on ice for 30 min. Then, samples (5–10 µg protein) were diluted 5:1 v/v in reducing sample buffer (0.25 m Tris, 10% w/v SDS, 30% v/v glycerol, 0.02% w/v bromophenol blue, 5% v/v *β*‐mercaptoethanol) and heated at 95 °C for 10 min. Proteins were resolved on 12% SDS polyacrylamide gels and transferred on 0.2 µm pore‐sized Immun‐Blot PVDF membranes. The membrane was blocked with 5% w/v skim milk powder dissolved in Tris buffered saline (TBS‐T, 20 × 10^−3^ m Tris, 150  × 10^−3^ m NaCl, 1% v/v polysorbate 20) for 2 h at RT. The membrane was probed with primary mouse monoclonal anti‐CD73, anti‐CD63, anti‐CD9, anti‐TSG101, anticalregulin, and anti‐GAPDH antibody for overnight at 4 °C. Then, the membrane was washed four times for 5 min each with TBS‐T and incubated with the secondary HRP‐conjugated polyclonal goat antimouse immunoglobulin for 2 h at RT. The membrane was again washed four times for 5 min each with TBS‐T and incubated with Western Blotting Luminol Reagent for 2 min at RT. Protein bands were developed on Fuji medical X‐ray films in an Agfa Curix 60 (Agfa Corporate, Mortsel, Belgium).

##### TEM

The exosome morphology was visualized by TEM as described elsewhere.^[^ ^23^ ^]^ Exosomes (2 µL) were blotted on glow‐discharged (30 s in an Emitech K100X glow discharge system, Quorum Technologies Ltd., Lewes, UK) carbon‐coated grids. Grids were washed with PBS for 2 min, fixed with 1% v/v glutaraldehyde for 5 min, and rinsed eight times with double‐distilled water for 2 min each. Excess liquid was drained off with filter paper. The sample was stained with uranyl oxalate at pH 7 for 5 min and then incubated in a mixture of 2% v/v methylcellulose and 4% v/v uranyl acetate for 10 min on ice. Excess liquid was drained off and the grids were air‐dried. The fixed sample was analyzed in a FEI Morgagni 268 microscope (Field Electron and Ion Company, Hillsboro, OR, USA) operated at a 100 kV acceleration voltage in the bright field mode. Size distributions from TEM images were obtained with FIJI software.^[^ ^69^ ^]^

##### Cryo‐TEM

Vitrified exosomes were analyzed by cryo‐TEM. The exosome suspension (3 µL) was added on glow‐discharged (30 s in an Emitech K100X glow discharge system, Quorum Technologies Ltd.) 300‐mesh lacey carbon‐coated copper grids. The sample was vitreously frozen in a mixture of liquid ethane and propane in a Vitrobot Mark II (Field Electron and Ion Company). Excess sample was removed by controlled blotting. Grids were mounted in a Gatan cryo‐holder and transferred into a Tecnai F20 Cryo (Field Electron and Ion Company). The microscope was operated at a 200 kV acceleration voltage in the bright field mode and maintained at −180 °C during sample analysis. Micrographs were recorded under low‐dose conditions (<500 electrons nm^−2^) using a Falcon II 4K Direct Electron Detector (Field Electron and Ion Company).

##### In Vitro Scratch Wound Healing Assay

The scratch wound healing assay was performed as previously reported.^[^ ^70^ ^]^ Immortalized human keratinocytes (HaCaT cells^[^ ^42^ ^]^) and spontaneously immortalized mouse keratinocytes were seeded at a density of 105 000 cells per well and primary human dermal fibroblasts at 30 000 cells per well in a 48‐well plate. Primary HUVEC were seeded at 50 000 cells per well in a 48‐well plate precoated with PureCol bovine collagen type I (50 µg mL^−1^). Cells were grown to a confluent monolayer and a cross‐shaped wound was scraped into it using a sterile 200 µL pipette tip. In some migration studies with HaCaT, cellular proliferation was inhibited by incubation with mitomycin C (2 µg mL^−1^) for 2 h prior to scratching. The cells were washed with PBS and treated with HS‐5 exosomes (10, 30, or 90 µg protein mL^−1^), heat‐inactivated HS‐5 exosomes (90 µg protein mL^−1^, 65 °C, 2.5 h), CD73‐inhibited HS‐5 exosomes (90 µg protein mL^−1^, 30 × 10^−6^ m APCP), SELL (1.3 × 10^11^ vesicles mL^−1^) or variations thereof, PC‐Chol liposomes (1.3 × 10^11^ vesicles mL^−1^), EGF (10 or 50 ng mL^−1^) or VEGF (20 ng mL^−1^), buffer control or APCP control. The concentrations refer to a volume of 150 µL per well. The wound was imaged with a Leica DMI6000 B epifluorescence microscope (Leica Microsystems, Wetzlar, Germany) at a 2.5× magnification after 0 and 24 h. The wound area was analyzed with FIJI software.^[^ ^69^ ^]^ The percentage of the wound closure was calculated and normalized to the medium control.

##### In Vitro Tube Formation Assay

The capillary tube formation assay was performed according to a prior published protocol,^[^ ^71^ ^]^ with minor modifications. Primary HUVEC were seeded at a density of 50 000 cells per well in a 48‐well plate precoated with PureCol bovine collagen type I (50 µg mL^−1^) and grown to confluency. Cells were washed with PBS and starved for 4 h in EBM‐2 (EBM supplemented with 2% v/v FBS, 100 U mL^−1^ penicillin, and 100 µg mL^−1^ streptomycin). HS‐5 exosomes (90 µg protein mL^−1^), heat‐inactivated HS‐5 exosomes (90 µg protein mL^−1^, 65 °C, 2.5 h), CD73‐inhibited HS‐5 exosomes (90 µg protein mL^−1^, 30 × 10^−6^ m APCP), SELL (1.3 × 10^11^ vesicles mL^−1^) or variations thereof, PC‐Chol liposomes (1.3 × 10^11^ vesicles mL^−1^), VEGF (20 ng mL^−1^), buffer or APCP control were suspended in EBM‐2 containing 1 mg mL^−1^ PureCol bovine collagen type I pH 7.4 and 400 µL were added per well. The tube formation was imaged with a Leica DMI6000 B epifluorescence microscope (Leica Microsystems) at a 2.5× magnification after 20 h. Images were analyzed with FIJI software applying the Skeletonize3D and AnalyzeSkeleton plugins.^[^ ^69^, ^72^, ^73^ ^]^

##### Sample Preparation for Quantitative Proteomics Analysis

For quantitative proteomics analysis, exosome samples were processed using the iST Kit according to the manufacturer's instructions (PreOmics GmbH, Planegg/Martinsried, Germany). In brief, exosome samples (20 µg) were diluted in “Lyse” buffer, boiled at 95 °C for 10 min and subsequently processed with high intensity focused ultrasound (30 s, 85% amplitude). Samples were loaded in the supplied cartridge and on‐filter digested with 50 µL “Digest” solution for 60 min at 37 °C. Then, 100 µL “Stop” solution was added and the cartridge was centrifuged at 3800 × *g*. Digested peptides were washed with 100 µL of each “Wash 1” and “Wash 2” solution, eluted in 100 µL “Elute” solution, dried, and resuspended in 20 µL “LC‐Load” buffer for mass spectrometric (MS) analysis.

##### Liquid Chromatography‐Mass Spectrometry (LC‐MS) Analysis for Quantitative Proteomics

In a randomized order, 3 µL per peptide sample was separated on a nanoEase M/Z Symmetry C18 Trap Column coupled to a nanoEase M/Z C18 HSS T3 Column by a 0.1% formic acid in water (solvent A) and 0.1% formic acid in acetonitrile (solvent B) gradient (from 8% to 22% B in 80 min, 32% B in 10 min, and 95% B in 1 min) at a flow rate of 0.3 mL min^−1^ using an M‐Class Ultra Performance LC (Waters Corporation). Separated peptides were then analyzed on‐line on an Orbitrap Fusion Lumos Tribrid mass spectrometer (Thermo Fisher Scientific) equipped with a Digital PicoView nanospray source (New Objective Inc., Woburn, MA, USA). Full‐scan MS spectra (300–1500 *m*/*z*) at a resolution of 120 000 at 200 *m*/*z*, and a target value of 500 000 charges per acquisition were recorded in the data‐dependent acquisition mode. Data‐dependent tandem MS spectra were recorded in the linear quadrupole ion trap operated in the rapid scan mode with a target value of 10 000 charges per acquisition, a 0.8 Da isolation width, a 50 ms maximum injection time, and a 35% higher energy collision induced dissociation fragmentation energy. Only precursor ions with an intensity above 5000 were selected for tandem MS, with the maximum cycle time set to 3 s and an enabled charge state screening. Singly, unassigned and charge states greater than seven were rejected. Precursor masses previously selected for tandem MS measurements were excluded from further selection for 30 s, and the exclusion window was set at 10 ppm. Real‐time calibration on an internal lock mass of 371.1012 and 445.1200 *m*/*z* was performed. The MS proteomics data was handled using the local laboratory information management system.^[^ ^74^ ^]^

##### MS Proteomics Data Analysis

The MS raw data was searched against the Swissprot *Homo sapiens* reference proteome (taxonomy 9606, version from 2019‐07‐09) and concatenated to its reversed decoyed fasta database and common protein contaminants using the integrated Andromeda search engine in MaxQuant (version 1.6.2.3).^[^ ^75^ ^]^ Cysteine carbamidomethylation and N‐terminal protein acetylation was set as either fixed or variable modification. Enzyme specificity was set to trypsin/P allowing a minimal peptide length of seven amino acids and a maximum of two missed‐cleavages. A false discovery rate (FDR) of 0.01 for peptides and 0.05 for proteins was used. Label free quantification intensities were log2 transformed and normalized. Statistical analysis was performed using the *R* environment for statistical computing with Bioconductor (version 3.6.2).^[^ ^76^ ^]^ Employing the *R* package limma (version 3.42.0), differentially abundant protein analysis was modeled using mixed effect linear regression with empirical Bayes variance estimation.^[^ ^77^ ^]^ Proteins involved in wound healing were searched and classified against the AmiGO 2 database (version 2.5.13).^[^ ^78^ ^]^ The protein accession code was based on UniProt.^[^ ^79^ ^]^

##### Lipidomics Analysis

For lipidomics analysis, exosomes were resuspended in methanol/water 4:1 v/v and centrifuged at 4 °C and 16 000 × *g* for 15 min. Exosomal lipids (1 µL of the supernatant) were separated on a Acquity UPLC HSS T3 Column using a 5 × 10^−3^ m ammonium acetate in water/acetonitrile 95:5 (A) and 5 × 10^−3^ m ammonium acetate in isopropanol/acetonitrile 90:10 (B) gradient (from 5% B to 100% B over 12 min) at an adjusted flow rate of 3–4 µL min^−1^ over 12 min using a nanoAcquity UPLC (Waters Corporation). Separated lipids were then analyzed on‐line on a Q Exactive Mass Spectrometer (Thermo Fisher Scientific) equipped with a nanoelectrospray ionization source. MS spectra (50–1200 *m*/*z*) were recorded at a MS resolution of 70 000 and MS/MS resolution of 17 500 using negative and positive polarization and all ion fragmentation. Lipid data sets were analyzed with Progenesis QI Software (Nonlinear Dynamics, Milford, MA, USA). Detected ions were identified based on accurate mass, adduct patterns, as well as isotope patterns by searching against the Lipid Maps Structure Database^[^ ^80^ ^]^ with a mass accuracy tolerance of 5 MDa, and the relative abundance of each lipid species was normalized.

##### In Vivo Wound Healing Studies

Animal care and experimental protocols had been approved by the local veterinary authorities (Kantonales Veterinäramt, Zurich, Switzerland) and were performed according to Swiss law. Four 5 mm full‐thickness excisional wounds were punched in the dorsum of anaesthetized female C57BL/6JRj mice at the age of 9 weeks,^[^ ^47^ ^]^ with 6 mice per treatment group. Immediately postwounding and then every second day, 50 µL of HS‐5 exosomes (15 µg proteins or 1.5 × 10^11^ vesicles), SELL (1.5 × 10^11^ vesicles), or PBS were applied by two intradermal injections (25 µL each) thereof at the wound edges. Wounds were allowed to heal without dressing and collected at day 5 post‐wounding for analysis.

##### Analysis of Skin Wounds by Histomorphometry and Immunofluorescence Staining

Wounds were excised and either fixed with ethanol/acetic acid 95:1 v/v overnight or 4% v/v paraformaldehyde in PBS followed by paraffin embedding, or directly frozen in tissue‐freezing medium. Sections (7 µm) from the middle of the wound were then processed for either histological or immunofluorescence analysis.

For histological analysis, sections were stained with H&E and according to the Herovici stain procedure^[^ ^81^ ^]^ and subsequently imaged with a Panoramic 250 Slide Scanner (3D Histech, Budapest, Hungary).

For immunohistochemistry analysis, paraffin sections were dewaxed, rehydrated in a xylene/ethanol gradient, and incubated in citrate buffer pH 6.0 for 1 h at 95 °C to retrieve antigens. Wound sections were blocked with PBS containing 12% w/v BSA for 1 h at RT and incubated with the primary rabbit monoclonal anti‐Ki67 antibody, followed by incubation with a biotinylated secondary anti‐rabbit antibody. For bright‐field analysis, bound antibodies were detected with the Vectastain ABC kit and the diaminobenidine peroxidase substrate kit according to the manufacturer´s instructions (Vector Laboratories). For immunofluorescence analysis, cryo‐wound sections were washed twice with PBS containing 0.1% v/v Triton X‐100 and subsequently fixed with cold acetone. Sections were washed and blocked with PBS containing 12% w/v BSA for 1 h at RT, followed by incubation with primary antibodies (anti‐Meca32, antiactin *α*‐SMA) for overnight at 4 °C. Sections were then incubated with the secondary Cy3 antimouse IgG antibody for 30 min at RT and counterstained with Hoechst 33342. Stained sections were imaged with an Axioskop 2 microscope equipped with a Plan‐Neofluar objective (20×/0.5NA) and photographed with an Axiocam HRc camera (all from Carl Zeiss Microscopy GmbH, Jena, Germany). The Axiovision 4.6 software (Carl Zeiss Microscopy GmbH) was used for data acquisition. Images were then analyzed using ImagePro Plus software (Media, Cybernetics Inc., Rockville, MD, USA).

##### Statistical Analysis

Statistical analysis was performed using GraphPad Prism (version 8.2.0, GraphPad Software Inc., San Diego, CA, USA). Data was presented as mean + SD, unless stated otherwise. For in vitro cell‐based assays (*n* = 3–5), relative abundance of CD73 (*n* = 4 per passage number) and CD73 activity (*n* = 3–4 per passage number), mean differences between groups were calculated with an ordinary two‐way ANOVA followed by a post‐hoc Tukey's multiple comparison test (*α* = 0.05, *p*‐value calculated), assuming a normal distribution of the data. For the in vivo studies, outliers were identified with the ROUT method (*Q* = 1%) and excluded from the analysis. Outliers (one value per treatment and control group) were only identified and removed in the Herovici analysis. Treatments were compared to the control (both *n* = 6–11 wounds analyzed from 6 mice per group) by an unpaired, non‐parametric Mann–Whitney *U* test (*α* = 0.05, *p*‐value calculated).

## Conflict of Interest

The authors declare no conflict of interest.

## Author Contributions

J.‐C.L. and B.F.H. designed the overall project and wrote the manuscript. B.F.H. performed the in vitro experiments and analyzed all data. J.‐C.L., S.W., M.B.‐Y.G., and B.F.H. planned the in vivo study. M.B.‐Y.G. conducted the in vivo study and analyzed the in vivo data. S.W. and M.B.‐Y.G. provided feedback on the manuscript.

## Supporting information

Supporting Information
