# Supplementary material for: Exosomes for Wound Healing: Purification Optimization and Identification of Bioactive Components
Source: Adv Sci (Weinh). 2020 Oct 27;7(23):2002596. doi: 10.1002/advs.202002596 (PMC7709981; doi:10.1002/advs.202002596)
Supplement: Supplementary file 1 — Supporting Information [file ADVS-7-2002596-s001.pdf]

WILEY-VCH

© 2020 Wiley-VCH GmbH

ADVANCED SCIENCE
Open Access

Supporting Information

for *Adv. Sci.*, DOI: 10.1002/advs.202002596

Exosomes for wound healing: purification optimization and identification of bioactive components

*Britta F. Hettich, Maya Ben-Yehuda Greenwald, Sabine Werner, and Jean-Christophe Leroux**

Supporting Information

Exosomes for wound healing: purification optimization and identification of bioactive components

*Britta F. Hettich, Maya Ben-Yehuda Greenwald, Sabine Werner, Jean-Christophe Leroux**

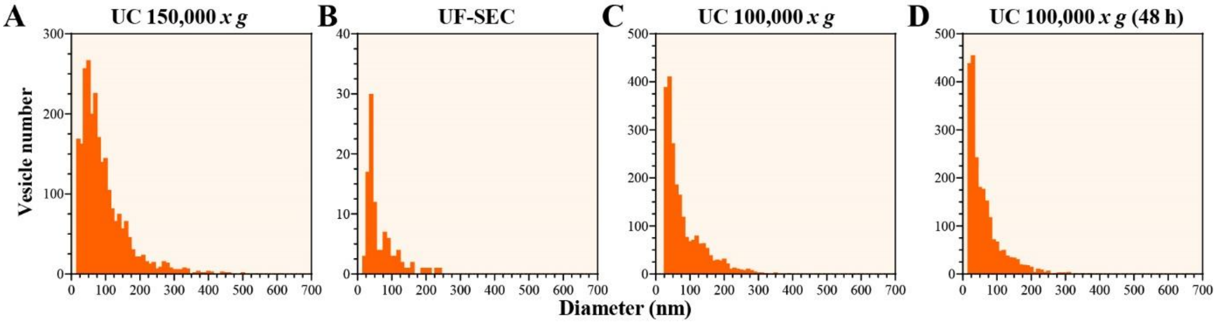

Figure S1. The purification process affects HS-5 exosome size distribution. (**A**-**D**) Exosomes were produced for 24 h (A-C) or 48 h (D) and isolated by UC at either 150,000 *x g* (A) or 100,000 *x g* (C, D) or by UF-SEC (B). Size distribution profiles were obtained from TEM images.

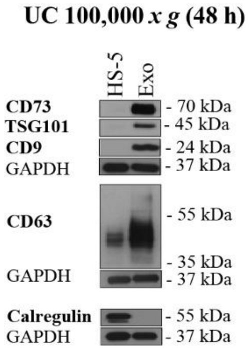

Figure S2. Exosome identity and purity following UC at 100,000 *x g*. Exosomes were produced for 48 h and isolated by UC at 100,000 *x g*. The exosome markers CD73, TSG101, CD9 and CD63 and the contamination marker calregulin were detected by immunoblotting. GAPDH served as loading control. Experiments were performed in triplicates and a representative image is shown.

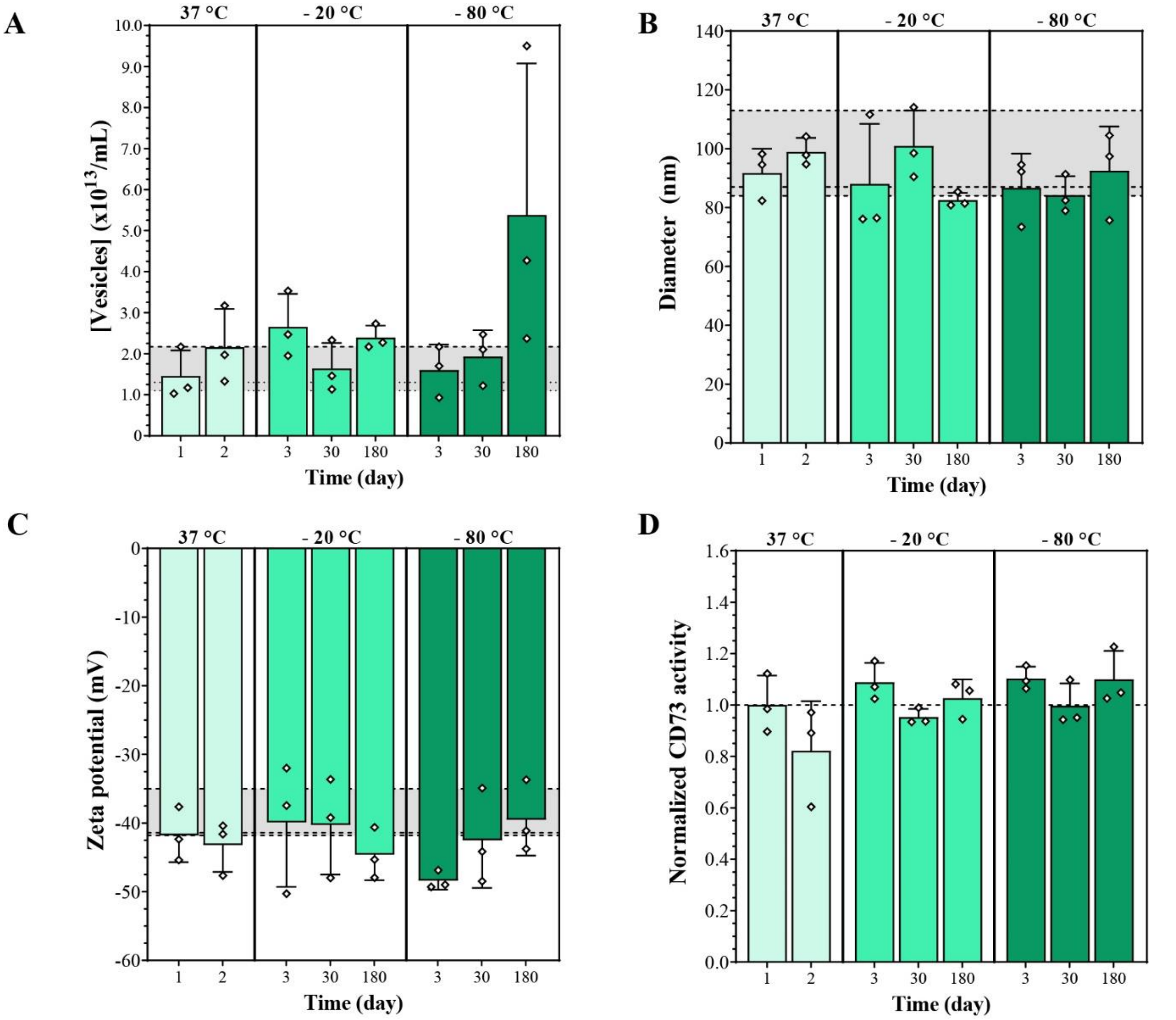

Figure S3. Storage conditions have no relevant impact on the HS-5 exosome characteristics. (**A-D**) Exosome suspensions were aliquoted and stored either at 37 °C for up to 48 h or at -20 °C or -80 °C for up to 6 months. The vesicle concentration (A), diameter (B) and zeta potential (C) were analyzed by NTA. The CD73 activity was determined by a customized malachite green assay and values are normalized to sample values at day zero (D). The dashed lines and/or the grey area mark the sample values at day zero. Data represent mean + SD, n = 3.

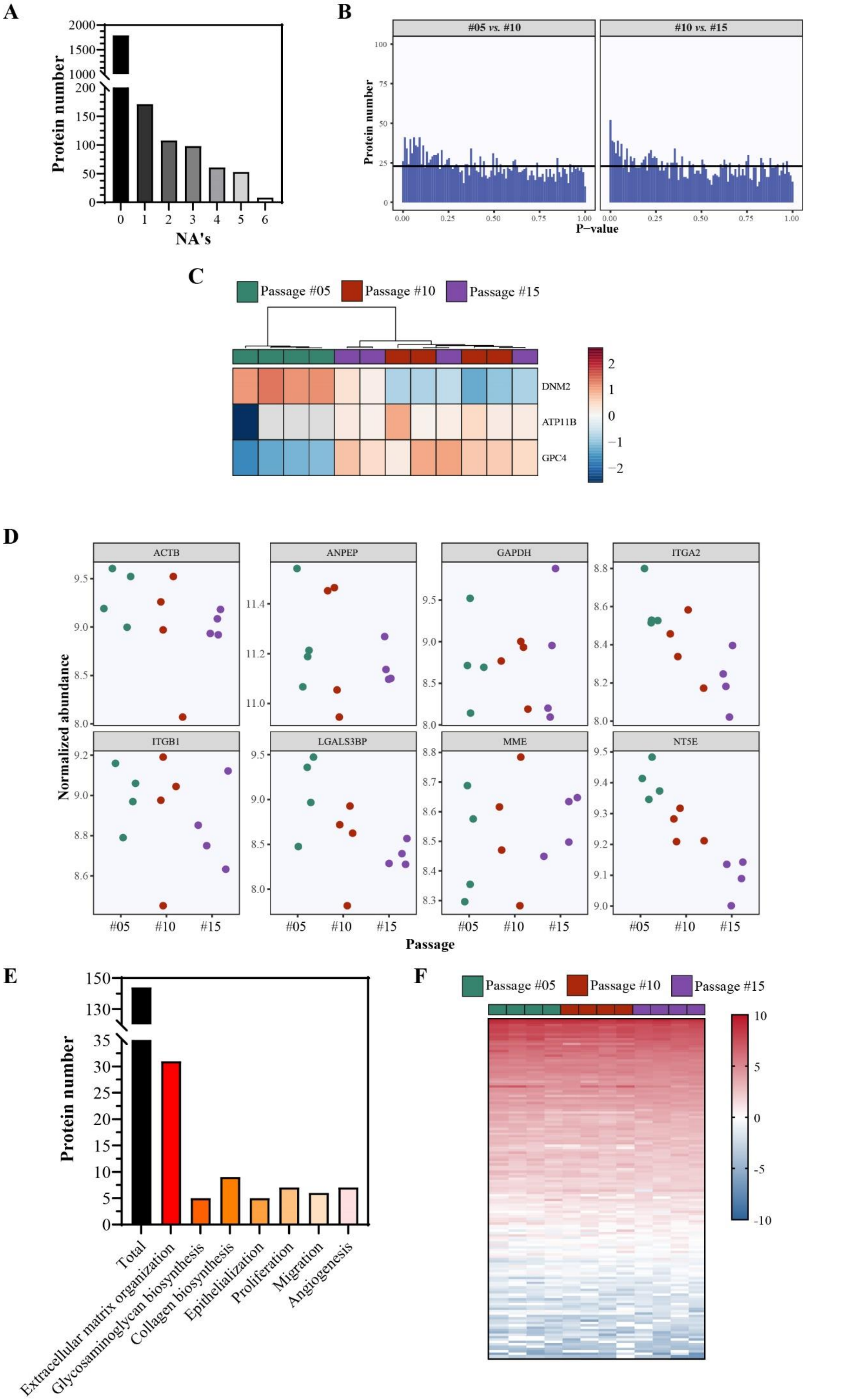
A
Protein number
NA's
2000
1500
1000
200
150
100
50
0
0 1 2 3 4 5 6
B
#05 vs. #10
#10 vs. #15
Protein number
P−value
C
Passage #05
Passage #10
Passage #15
DNM2
ATP11B
GPC4
D
ACTB
ANPEP
GAPDH
ITGA2
ITGB1
LGALS3BP
MME
NT5E
Normalized abundance
Passage
#05 #10 #15
E
Protein number
Total
Extracellular matrix organization
Glycosaminoglycan biosynthesis
Collagen biosynthesis
Epithelialization
Proliferation
Migration
Angiogenesis
F
Passage #05
Passage #10
Passage #15
10
5
0
-5
-10

Figure S4. HS-5 cells between passages #05 and #15 produce exosomes with similar protein composition. (**A**) Number of proteins (y-axis) that could not be assigned in n exosome replicates (NA's, x-axis). n = 4 per passage number. (**B**) Distribution profiles of the p-values comparing exosomes from passages #05 *vs.* #10 (left panel) and passages #10 *vs.* #15 (right panel). (**C**) Heat map and hierarchical clustering of the protein levels of the top three differentially abundant proteins at a FDR of 0.05 *vs.* sample matrix. The color key indicates the log2 fold-change, comparing either passage #05 *vs.* #10 or #10 *vs.* #15. Darker blue colors correspond to relative downregulation, darker red color to relative upregulation. n = 4 per passage number. (**D**) The normalized protein levels (y-axis) of the top eight abundant proteins is shown as a function of cell passage (x-axis). Different colors represent the different passages, while independent replicates of the same conditions have the same color. n = 4 per passage number. (**E, F**) Proteins with a documented function in wound healing. Protein number classified based on the biological function (E). Heat map of protein levels of all wound healing-related proteins *vs.* sample matrix (F). The color key indicates the normalized abundance. Darker blue colors correspond to low abundance, darker red colors to high abundance. n = 4 per passage number.

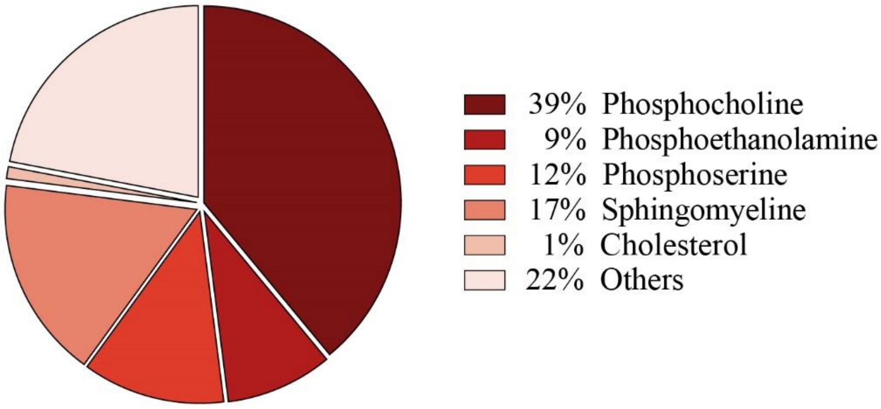

Figure S5. Lipidomic characterization of HS-5 exosomes. Overview of the different lipid classes identified in HS-5 exosomes by LC-MS analysis. Other lipids include ceramides, diacylglycerol, phosphatidylinositol and phosphatidic acids. Data represent relative percentage of the total lipid quantity and was averaged from three independent measurements.

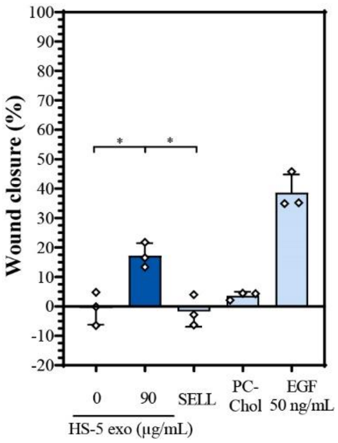

Figure S6. Purified HS-5 exosomes have a low pro-migratory activity on HaCaT *in vitro*. Cellular proliferation was inhibited by mitomycin C (2µg/mL, 2 h). After scratch wounding, cells were treated with HS-5 exosomes (90 µg protein/mL), SELL ($1.3*10^{11}$ vesicles/mL), PC-Chol liposomes ($1.3*10^{11}$ vesicles/mL) or the positive control EGF (50 ng/mL). The percentage of the wound closure was analyzed 24 h after scratch wounding and normalized to the medium control. Data represent mean + SD, n = 3. Significance was calculated with an ordinary two-way ANOVA followed by a post-hoc Tukey's multiple comparison test, $*p < 0.05$.

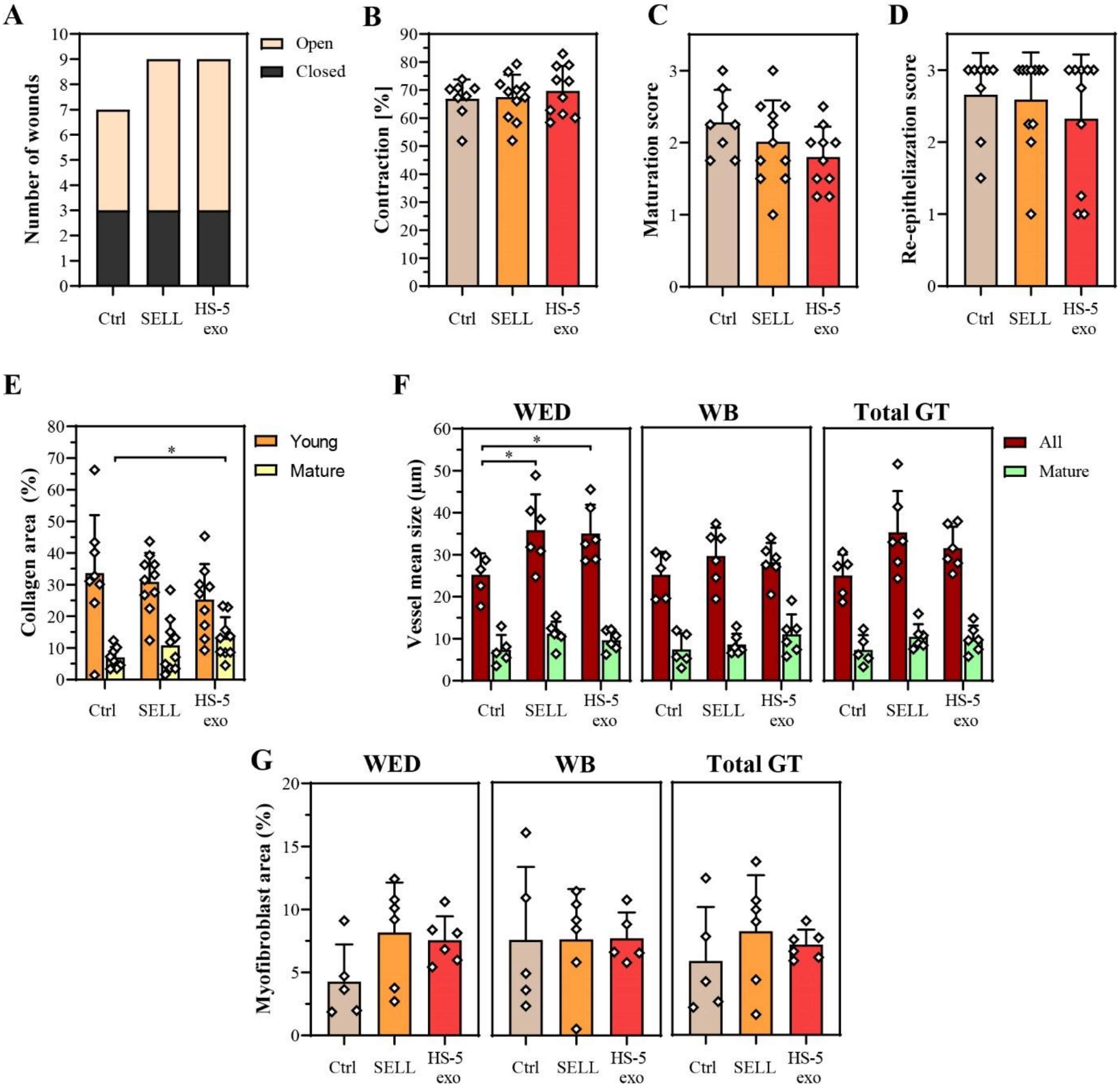

Figure S7. Effect of HS-5 exosomes on skin wound healing. (**A-D**) Histomorphometric wound parameters. Number of open *vs.* closed wounds (A) and wound contraction (B). Semi-quantitative wound scoring (0-3 ± 0.5) performed in a blinded fashion based on H&E-stained wound sections from day 5 post-wounding to assess maturation of the granulation tissue (C) and re-epithelialization (D). (**E**) Young and mature collagen expressed as percentage of the GT area. (**F**) Mean sizes of all and mature blood vessels in the WED, WB and total GT. (**G**) Area covered by myofibroblasts, which stained positive for α-SMA and negative for Meca32 in the WED, WB and total GT. Data (A-G) represent mean + SD, n = 5-11 wounds from 6 mice per treatment group. Significance was calculated with an unpaired, non-parametric Mann-Whitney U test, *p <0.05.

Table S1. Summary of the main functions of the differentially abundant proteins DNM2, ATP11B and GPC-4.

UniProt	Protein name	Main function
P50570	DNM2 (Dynamin-2)	Regulation of endocytosis and membrane fusion
Q9Y2G3	ATP11B (Probable phospholipid transporting ATPase IF)	Component of the P4-ATPase-flippase complex catalyzing ATP hydrolysis
O75487	GPC4 (Glypican-4)	Cell surface proteoglycan likely involved in cellular uptake of vesicles

Table S2. HS-5 exosomal proteins with a documented role in wound healing. HS-5 exosomal proteins were identified by quantitative proteomics and classified for their function in proliferation (P), migration (M), epithelialization (E), collagen biosynthesis (C), glycosaminoglycan biosynthesis (G), angiogenesis (A) and extracellular matrix organization (O). The most abundant proteins for each function are shown.

UniProt	Protein name	Main function
P00533	EGFR (Epidermal growth factor receptor)	E, M, P
P02452	COL1A1 (Collagen alpha-1(I) chain)	A, C, M, O
P02751	FN (Fibronectin)	A, O, P
P05106	ITGB3 (Integrin beta-3)	A, M, O
P05121	PAI-1 (Plasminogen activator inhibitor 1)	A, E, M, O
P05231	IL6 (Interleukin-6)	A, C, E, O
P05556	ITGB1 (Integrin beta-1)	A, E, M, O
P07355	ANXA2 (Annexin A2)	A, P, O
P07585	PG-S2 (Decorin)	G, O
P07996	TSP1 (Thrombospondin-1)	A, M, O, P
P08123	COL1A2 (Collagen alpha-2(I) chain)	A, C, M, O
P08648	ITGA5 (Integrin alpha-5)	A, E, M, O
P08670	VIM (Vimentin)	C, P
P09038	FGF2 (Fibroblast growth factor 2)	A, M, O, P
P09619	PGFRB (Platelet-derived growth factor receptor beta)	C, G, M, O, P
P15291	B4GALT1 (Beta-1,4-galactosyltransferase 1)	A, G, O
P16070	CD44 (CD44 antigen)	M, O
P16234	PDGFRA (Platelet-derived growth factor receptor alpha)	M, O, P
P17301	ITGA2 (Integrin alpha-2)	C, O, P
P17813	ENG (Endoglin)	A, C, O, P
P21580	TNFAIP3 (Tumor necrosis factor alpha-induced protein 3)	A
P23142	FBLN1 (Fibulin-1)	O, P
P24821	TNC (Tenascin-C)	O
P31431	SDC4 (Syndecan-4)	A, G, M
P41221	WNT5A (Protein Wnt-5a)	A, M, P
P42345	mTOR (Serine/threonine-protein kinase mTOR)	E, M, P
P55011	SLC12A2 (Solute carrier family 12 member 2)	A
P78536	ADAM17 (Disintegrin and metalloproteinase domain-containing protein 17)	E, M, P
Q03135	CAV1 (Caveolin-1)	E, M, O, P
Q12884	FAP (Prolyl endopeptidase FAP)	O
Q13418	ILK (Integrin-linked protein kinase)	A, E, M, P
Q9Y624	JAM-A1 (Junctional adhesion molecule A1)	A, O

Table S3. CD73 inactivation can be accomplished by heat or APCP treatment. Exosomes were heated at 65 °C for 2.5 h or simultaneously treated with the CD73 inhibitor APCP (30 µM). The CD73 activity was determined by a malachite green assay at time zero and after 24-h incubation at 37 °C to ensure irreversibility of CD73 inactivation. Data represent mean ± SD from three independent measurements.

Time [h]	Untreated [U/mg]	65 °C [U/mg]	APCP [U/mg]
0	4.5 ± 0.1	0	0
24	4.2 ± 0.2	0	0

Table S4. Different liposome formulations have a similar modal diameter. Liposomes were prepared by the thin-film hydration method and their size was analyzed by NTA. Data represent mean ± SD from three independent measurements.

Liposome formulation	Modal diameter [nm]
PC-Chol	85 ± 6
SELL	82 ± 3
SELL - no SM	88 ± 5
SELL - no DOPS	86 ± 7
SELL - no DOPE	78 ± 1
